# Effect of ficin‐hydrolyzed wheat gluten on bread quality and in vitro antioxidant activity before and after simulated gastrointestinal digestion

**DOI:** 10.1002/fsn3.3871

**Published:** 2023-11-27

**Authors:** Mojan Seyedain‐Ardabili, Mohammad‐Hossein Azizi

**Affiliations:** ^1^ Department of Food Science & Technology, Faculty of Agriculture Tarbiat Modares University Tehran Iran

**Keywords:** antioxidant, bread, ficin, functional foods, simulated digestion, wheat gluten hydrolysate

## Abstract

This study aimed to investigate the effect of adding ficin‐hydrolyzed wheat gluten at different levels (0%, 1%, 2%, 4%) on bread quality, and in vitro antioxidant activity before and after simulated gastrointestinal digestion. Our findings revealed that the incorporation of the generated wheat gluten hydrolysates (WGH) up to 4 g per 100 g flour positively affected the technological and physical–chemical characterizations of breads, including dough rheological properties, color, specific volume, and moisture. The texture profile analysis indicated reductions in hardness, springiness, and chewiness of the breads, and confirmed anti‐staling properties during storage. The enriched breads received satisfactory scores from the sensory panel and were perceived as less stale after a 4‐day period of storage. The aroma score of the 4% WGH bread was significantly higher than other treatments. Regarding taste, the 4% WGH bread scored the lowest, but the obtained value was not statistically significant. The enriched breads exhibited DPPH, ABTS radical scavenging, and Fe^2+^ chelation abilities that increased in response to higher levels of hydrolysate incorporation, and these antioxidant activities were enhanced after simulated gastrointestinal digestion. Our findings confirm that it is possible to apply ficin‐generated WGH to enhance physicochemical, nutritional, and biological quality of bread.

## Graphical abstract

Our study investigated the effect of adding ficin‐hydrolyzed wheat gluten at different levels (0%, 1%, 2%, 4%) on bread quality, and in vitro antioxidant activity before and after simulated gastrointestinal digestion. Ficin‐hydrolyzed gluten up to 4 g per 100 g flour positively influenced bread technological and physical‐chemical characterizations, and exhibited anti‐staling properties. The enriched breads demonstrated antioxidant abilities that increased in response to higher levels of hydrolysate inclusion and were enhanced by simulated gastrointestinal digestion.

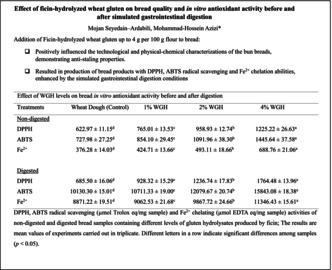

## 1 INTRODUCTION

In many countries, bread is a staple food and accounts for a large portion of a standard diet, providing a significant amount of energy, carbohydrates, proteins, and micronutrients. While bread has not always been in the form we know today, it has been an important part of our daily diet for centuries. In recent years, the food industry has focused on innovative approaches in meeting consumer demands, to satisfy the increased interest in food with health benefits (Altamirano‐Fortoul et al., 2012; Udomkun et al., 2022). Bread made from refined wheat flour, a common ingredient in bakery products, is less nutritionally balanced and has deficiencies such as low antioxidant activity. To enhance the functional properties of wheat flour proteins, optional ingredients may be added. To control the quality of food and diseases, lipid peroxidation in foods and the generation of free radicals in the body must be prevented, as free radicals are linked to various illnesses such as heart disease, hypertension, and even cancer (Shahi et al., 2020; Zhao et al., 2021). Due to the health risks and restrictions associated with synthetic antioxidants, protein hydrolysates have gained attention as natural antioxidants. Biologically active hydrolysates and peptides possess diverse structures, amino acid compositions, and sequences that determine their functions in food, cosmetics, and pharmaceutical products (Akbarmehr et al., 2023; Madruga et al., 2020). The antioxidant activity of proteins is suppressed if free radical scavenging amino acids are buried within the protein, making them inaccessible to pro‐oxidants. When performed under controlled conditions, enzymatic hydrolysis exposes antioxidant amino acids in proteins, resulting in hydrolysates with free radical scavenging properties (Franco‐Miranda et al., 2017).

As a plant protein source, wheat gluten contains two main components, glutenins and gliadins, which are highly polymorphic polypeptides that include over 60 different molecular weight species with a relative molecular mass (Mr) ranging between 30,000 and 90,000 kDa (Zouari Ellouzi et al., 2014). In an effort to extend wheat gluten's utilization, much research has been conducted on enhancing functional properties such as solubility, emulsifying, and foaming capability (Drago & Gonzalez, 2001; Elmalimadi, Stefanovića, et al., 2017; He et al., 2019; Takeda et al., 2001; Wang et al., 2006; Zouari Ellouzi et al., 2014). Nowadays, considering the increasing supplies of this protein on the worldwide market, its relatively low price, and the expansion of wheat starch production, wheat gluten hydrolysis prospects are changing (Song et al., 2020; Wang et al., 2007). The structural modification of wheat gluten using proteolytic enzymes is an efficient protein modification method to change functionality and generate biologically active hydrolysates and peptides that could be used as bread improvers (Drago & Gonzalez, 2001; Zouari Ellouzi et al., 2014).

Ficin (EC 3.4.22.3), which is found in the latex, fruit, and leaves of fig trees (*Ficus carica* species), is among the most promising plant proteases. As a cysteine endopeptidase similar to papain and bromelain, ficin can be used to produce protein hydrolysates for various food applications. In addition to its high activity at pH 7, ficin is completely inactive at pH 3, suggesting that it is safe for consumption. Additionally, this protease is metabolized along with other proteins in food after hydrolysis (Aider, 2021). Furthermore, when producing food products with health‐promoting properties, it is important to investigate the bioactivity and stability of the added bioactive components after gastrointestinal digestion. In vitro digestion models are fast, safe, and without ethical restrictions, commonly conducted in order to simulate gastrointestinal conditions of the human body and to assess changes in bio‐accessibility and bio‐functionality (Zhang et al., 2022). It was the purpose of this study to explore how adding wheat gluten hydrolysate at different levels affects dough rheology, bread physicochemical properties, baking performance, and sensory quality when manufacturing pan bread. It also aimed to examine the antioxidant activity of the hydrolysate‐containing breads both before and after simulated in vitro gastrointestinal (GI) digestion.

## 2 MATERIALS AND METHODS

### 2.1 Materials

The commercial wheat gluten (81% ± 0.009 protein content (based on dry matter), 1.4% ± 0.002 ash, 1.4% ± 0.003 fat, and 5.6% ± 0.02 moisture content) was obtained from Ardineh Esfahan. Chemical materials including trolox (6‐hydroxy‐2,5,7,8‐tetramethylchroman‐ 2‐carboxylic acid), 2,2′‐azino‐bis (3‐ethylbenzthiazoline‐6‐sulfonic acid) diammonium salt) (ABTS), 2,2‐Diphenyl‐1‐picrylhydrazyl (DPPH), ferrozine (3‐(2‐Pyridyl)‐5,6‐diphenyl‐1,2,4‐triazine‐4′,4′′‐disulfonic acid sodium salt), along with biological substances including Ficin enzyme (EC 3.4.22.3, with the activity of ≥0.1 unit/mg solid), Amyloglucosidase (*A. niger*, A7095, ≥260 U/mL), Pancreatin (porcine pancreas, P1750, 4 × USP), and Pepsin (porcine gastric mucosa, P 7000, 2500 U/mL) were all procured from Sigma‐Aldrich company. Basic ingredients for the formulation of the white pan bread were acquired from local markets and bakery shops in Tehran. The same batch of wheat flour (extraction rate 72%) was used throughout the research. All chemicals and reagents used in this work were food‐grade or reagent‐grade.

### 2.2 Preparation of ficin‐hydrolyzed wheat gluten

Hydrolysis of the wheat gluten was done with ficin protease following the method described by Karimi et al. (2021) and Wang et al. (2007) with minor modifications, until a degree of hydrolysis (DH) of 7% was reached, monitored by the pH‐stat method of Adler‐Nissen (1986). The substrate was prepared by dispersing wheat gluten in distilled water (5% w/v). The aqueous gluten dispersion was heated for 10 min in a water bath at 85°C prior to enzymatic hydrolysis and then was rapidly cooled to the appropriate temperature. The ficin enzyme was added to the suspension based on the protein content of the wheat gluten using the enzyme‐to‐substrate ratio of 1:20 (w/w), and digestion with the enzyme was carried out for 3 h at the optimal condition (pH 7.0 at 37°C and under constant agitation at 200 rpm). In order to stop the hydrolysis, the enzyme was inactivated by heat treatment at 90°C for 15 min and centrifuged at 12,000 *g* for 10 min to remove the insoluble portion and collect the supernatant. The obtained hydrolysate was rapidly cooled to 25°C, and then freeze‐dried and stored at −20°C as wheat gluten hydrolysate (WGH) until use.

### 2.3 Sodium dodecyl sulfate‐polyacrylamide gel electrophoresis (SDS‐PAGE)

SDS‐PAGE was performed in order to examine molecular mass distribution following hydrolysis with ficin enzyme, and protein bands were identified by applying the standard protocol described by Schägger and von Jagow (1987) using 15% acrylamide gels.

### 2.4 Preparation of bun breads enriched with different levels of WGH

The following formula was used to prepare the bun breads: sugar (3 g), fat (3 g), salt (1 g), fresh yeast (5 g), wheat flour (100 g), and water (according to farinograph results) (Seyedain Ardabili et al., 2016). WGH was added to the flour at the levels of 0%, 1%, 2%, and 4%, and ingredients were mixed for 10 min with medium speed (rpm) to form the dough. Initial proofing was carried out for 25 min at 27°C with 70% relative humidity. Each dough sample was then rounded, shaped, and divided into 50 g pieces. Final proofing was conducted at 37°C with a relative humidity of 85% for 45 min. Afterward, during the baking stage, buns were exposed to a temperature of 180°C for 15 min using a rotary oven. As a final step, the bread samples were then cooled to room temperature, placed in polypropylene zip bags, and analyzed after 1 and 4 days.

### 2.5 Technological and physical–chemical characterization of breads

#### 2.5.1 Dough rheological properties by farinograph

In line with the American Association of Cereal Chemists' (AACC) approved Method 54–21, the following parameters were studied to determine the rheological characteristics of the doughs: dough development time (DDT), stability, mixing tolerance index (MTI), and water absorption (WA).

#### 2.5.2 Specific volume, moisture content, and color analysis

The volumes of the bread samples were determined following the AACC method, 10‐05.01 (Rapeseed Displacement). The specific volume was calculated as volume‐to‐mass ratio and expressed as mL/g. The moisture content of the breadcrumbs was evaluated at days 1 and 4 of storage in accordance with the AACC method 44‐15.02 (Air‐Oven). Effect of WGH on the color values of the breads (crumb and crust) was evaluated using a colorimeter (Color Flex, Hunter Lab Inc. USA) (Karimi et al., 2021). Three color parameters were measured including L* (− black to + white), a* (− green to + red), and b* (−blue to + yellow). The colorimeter device was calibrated using a white standard tile, and triplicate analysis was performed.

#### 2.5.3 Texture profile analysis

The texture profile analysis (TPA) of the bread samples was performed according to the method of Karimi et al. (2021) using a Texture Analyzer (Brookfield TPA, model CT3, USA). The hardness, cohesiveness, springiness, and chewiness parameters were recorded. To this end, breadcrumbs were cut into 20 × 20 × 20 mm cubes slices, and the compression (40%) of the breadcrumbs was performed using a TA‐4 cylindrical probe with a size of 38.1 mm diameter and a length of 20 mm with a force of 0.098 N. The trigger force was 0.049 N, and the test speed was 1.7 mm/s. In the post‐baking period, the TPA was done on days 1 and 4.

### 2.6 Evaluation of in vitro antioxidant activity

#### 2.6.1 Preparation of bread extracts

One gram of freeze‐dried breadcrumb powder was extracted for 1 h using a solution of sodium phosphate buffer saline (pH 7). Afterward, centrifugation at 12,000 *g* for 15 min was used to separate the extracts, and the resulting supernatants were stored in darkness at −20°C (Gawlik‐Dziki et al., 2013; Karimi et al., 2021).

#### 2.6.2 DPPH radical scavenging assay

The DPPH radical scavenging activity (RSA) of the bread extracts was performed according to previously described methods (Karimi et al., 2021; Seyedain‐Ardabili et al., 2023). Bread extract solutions (500 μL) were mixed 1:1 (v/v) with DPPH solution prepared in methanol (0.1 mM). The mixtures were vigorously shaken before being placed at room temperature for 30 min in a dark area. Afterward, the absorbance of the blank and sample solutions was determined at 517 nm with a UV–visible spectrophotometer (Agilent‐Carry 60). The results were expressed as μmol trolox/mg of samples by using trolox (20–200 μmol/L) as standard.

#### 2.6.3 ABTS^+^ radical scavenging assay

The ABTS RSA was evaluated according to previous literature (Franco‐Miranda et al., 2017; Seyedain‐Ardabili et al., 2023). The Bread extract solutions (20 μL) were mixed with 980 μL of an ABTS solution prepared in advance (absorbance of 0.700 ± 0.01 at 734 nm). The resulting mixtures were shaken and then incubated in a dark place at room temperature for 10 min. Absorbance for the blank and samples was read at 734 nm by UV spectrophotometer. The results were converted to μmol trolox equivalents per mg of samples using trolox standard with concentrations ranging from 50 to 1100 μM.

#### 2.6.4 Ferrous (Fe^2+^) chelating ability

The ferrous chelating capacity of the bread extracts was analyzed following previously described methods (Karimi et al., 2021; Seyedain‐Ardabili et al., 2023). The extracts (500 μL) were mixed with 1850 μL of distilled water and 50 μL of FeCl_2_ (2 mM) and were left at room temperature for 3 min. Next, ferrozine aqueous solution (100 μL, 5 mM) was added to the mixture and was left in the darkness to react for 20 min. The absorbance of the obtained complex and the control were read at 562 nm. The results were calculated as EDTA equivalents/mg sample by using an EDTA standard curve (10–80 mg/L).

#### 2.6.5 Digestion of the breads (in vitro)

In order to simulate gastric digestion conditions, dried bread samples (2.5 g) were added to 30 mL of distilled water, followed by 0.8 mL of 1 M HCl (pH 2.5). Next, a 10% pepsin solution (1 mL) was prepared in 0.05 M HCl and added to the bread sample mixtures, followed by incubation at 37°C for 30 min (constant stirring at 130 rpm). Then, the pH was adjusted to 6.0 using 2 mL of 1 M NaHCO_3_ and 5 mL of 0.1 M sodium phosphate buffer. Afterward, a 100 μL of amyloglucosidase (1% (v/v) solution in 0.1 mol/L sodium acetate buffer, pH 5.2) was added, immediately followed by the addition of a 5% pancreatin solution (5 mL) in 0.1 M sodium phosphate buffer (pH 6.0). The final volume was adjusted to 55 mL by adding distilled water, and in vitro digestion was carried out at 37°C for 120 min (slow continuous stirring at 130 rpm) (Karimi et al., 2021; Woolnough et al., 2010). The bread suspensions acquired after 120 min of simulated digestion were centrifuged at 12,000 *g* for 10 min. The supernatant was collected, freeze‐dried, and stored at −20°C. Radical scavenging activity (DPPH and ABTS) and Fe^2+^ chelating ability were investigated in accordance with the methods mentioned in above sections.

#### 2.6.6 Sensory evaluation

Sensory evaluation of the bread samples enriched with WGH was conducted by 12 trained panelists (5 males and 7 females, ranging in age from 30 to 50 years). All samples were coded with random and balanced three‐digit numbers, and assessed at room temperature. External characteristics (i.e., volume, color of crust, symmetry of form, evenness of bake, character of crust, break, and shred) and internal characteristics (i.e., grain, color of crumb, aroma, taste, chewability, and texture) were evaluated at day 1 of storage. To this end, perfect score method was used, according to the reports provided by the American Institute of Baking and previous literature (Karimi et al., 2021; Matz, 1972). The staleness of the bun breads was also evaluated in accordance with the AACC Method 74‐30.01 at days 1 and 4 of the storage period (at room temperature), using a defined rating scale with various degrees of perceived staleness (1 = very fresh, 6 = very stale).

### 2.7 Statistical analysis

Data were analyzed using one‐way analysis of variance (ANOVA) with JMP 10 statistical software, and both the student's *t*‐test and Tukey's test were used to assess the significant differences among the samples. The results were shown as means ± standard deviation (SD), and *p* < .05 was considered statistically significant.

## 3 RESULTS AND DISCUSSION

### 3.1 Enzymatic hydrolysis

According to research, the rate, extent, and pattern of gluten proteolysis may vary with gluten concentration, and more intensive proteolysis has been observed in more diluted suspensions (Elmalimadi, Jovanovića, et al., 2017). On the basis of these results, hydrolysis of gluten was performed using a 5% (w/v) concentration of gluten. Initial tests revealed that the enzyme–to‐substrate ratio (E: S) of 1:20 w/w was effective for gluten hydrolysis (data not shown). It was found that the optimal pH and temperature for ficin protease activity on wheat gluten were 7.0 and 37°C, respectively, in accordance with the range suggested by the manufacturer (Sigma‐Aldrich). The degree of hydrolysis (DH) is a measurement of the extent of hydrolysis and an important factor that affects the properties of hydrolysates, such as the amount of intact proteins, molecular weight distribution, and amino acid profile, further influencing the functional properties of hydrolysates (Elmalimadi, Jovanovića, et al., 2017; Elmalimadi, Stefanovića, et al., 2017; He et al., 2019).

Researchers have observed that native gluten displays poor water dispersibility and normally forms aggregates in water, and the low solubility of gluten at neutral pH might contribute to lower degrees of hydrolysis, which is more evident at higher gluten concentrations, but heat treatment significantly improves the susceptibility of gluten hydrolysates to enzymes. Also, the hydrolysis of gluten is strongly affected by the agitation speed (Elmalimadi, Stefanovića, et al., 2017; Takeda et al., 2001); therefore, in this research, wheat gluten was subjected to a thermal process at 85°C for 10 min prior to enzymatic hydrolysis, and agitation speed was maintained at 200 rpm. During the first 60 min of the reaction, the hydrolysis rate increased rapidly, and then slowly increased for the next 120 min, until the reaction reached a steady state (180 min). The DH value obtained for the hydrolysate sample collected at 180 min was 7 ± 0.1%. The endoprotease activity of ficin might also be responsible for this medium value of DH (Aider, 2021). Higher values of DH are often linked with more essential amino acids and increased nutritional value, whereas low DH values have the potential to provide functional properties such as foaming stability. Under these experimental conditions, it was possible to obtain a higher DH value, directly dependent on the enzyme concentration and proteolysis time and inversely dependent on the substrate concentration (Zapata‐Montoya et al., 2018).

### 3.2 Sodium dodecyl sulfate‐polyacrylamide gel electrophoresis (SDS‐PAGE)

**FIGURE 1:**
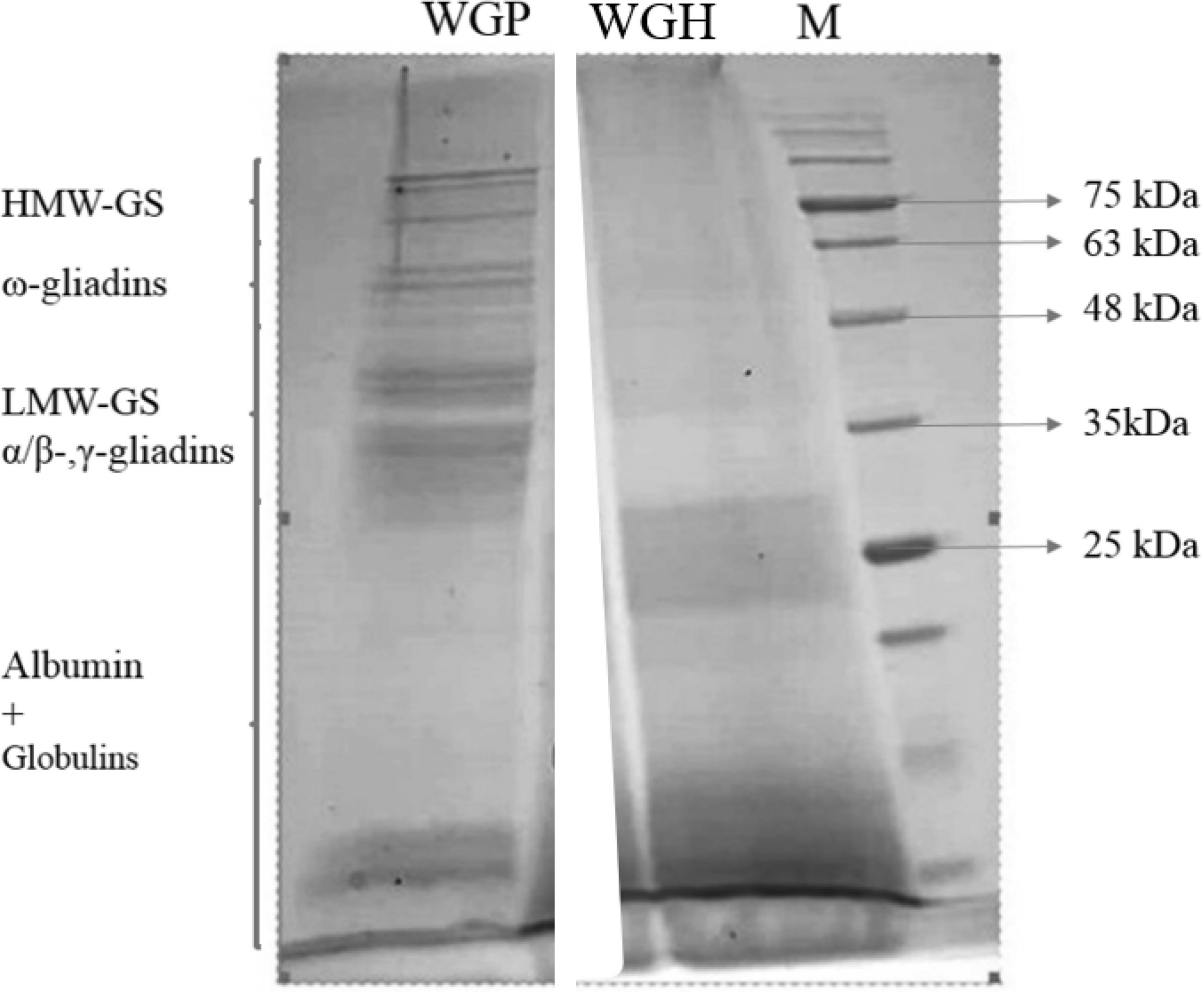
SDS‐PAGE profile of wheat gluten protein sample (WGP) and the hydrolysate sample (WGH). HMW‐GS, high‐molecular‐weight glutenin subunits; LMW‐GS, low‐molecular‐weight glutenin subunits; M, molecular mass marker.

The untreated wheat gluten and the resultant hydrolysate sample were analyzed using SDS‐PAGE to study changes in the molecular mass distribution after hydrolysis with ficin enzyme. According to research, gluten proteins are composed of glutenin and gliadin fractions with varying relative molecular weights (MW), with the glutenin subunits ranging from 30–50 kDa (LMW‐GS) to 65–90 kDa (HMW‐GS), and the gliadins (α, β, γ, and ω‐gliadins) ranging from 28 to 55 kDa. In general, the control wheat gluten sample displayed characteristic protein bands of various gluten protein fractions (Figure 1), with significant bands ranging from 25 to 85 kDa. A protein band at around 15 kDa was observed in the control sample, which could be due to the presence of albumin and globulin residues (Gabler & Scherf, 2020; Pourmohammadi & Abedi, 2021). After 180 min of hydrolysis with ficin protease, a significant reduction of bands corresponding to high‐molecular‐weight glutenins and gliadins was noted (MW ≥35 kDa), and major protein bands could be observed in the low‐molecular‐weight region of the obtained hydrolysate sample (WGH), at around 25 kDa and MW ≤15 kDa.

### 3.3 Effect of gluten hydrolysates on dough mixing properties.

The addition of WGH at different levels resulted in differences in dough mixing behavior and water absorption measured by the Farinograph (Table 1). Water absorption (WA) (water content necessary to obtain dough consistency of 500 BU) was modified by the incorporation of the hydrolysates above 1%, and the highest WA was observed in the dough containing 4% WGH, followed by the dough with 2% WGH. A similar trend was observed regarding dough development time (DDT) (the time required for reaching maximum consistency), and hydrolysate levels above 1% significantly increased DDT, with no significant differences between the control and the dough with 1% WGH. Regarding stability (time during which 500 BU dough consistency is maintained), only the value obtained for the dough with 4% WGH was significantly increased, in comparison with the other treatments and the control dough. Furthermore, WGH at 2 and 4% levels, significantly decreased the mixing tolerance index (MTI) (the difference in consistency between the highest peak and the peak after 5 min).

**TABLE 1:** Farinograph analysis of wheat dough containing WGH at different levels.

	Dough development time/min	Stability/min	Mixing tolerance index/BU	Water absorption
Wheat Dough	4.1 ± 0.2^a^	5.1 ± 0.2^a^	95 ± 2.4^a^	59.2 ± 0.2^a^
1% WGH	4.4 ± 0.3^a^	5.2 ± 0.1^a^	92 ± 1.0^a^	60.6 ± 0.7^a^
2% WGH	5.1 ± 0.2^b^	5.3 ± 0.2^a^	90 ± 1.0^b^	64.6 ± 1.0^b^
4% WGH	5.9 ± 0.1^c^	5.8 ± 0.1^b^	81 ± 2.0^c^	69.5 ± 1.3^c^

*Note*: The results are mean values of experiments carried out in triplicate. Different letters in a column indicate significant differences among samples (*p* < .05).

The rheological properties of wheat flour dough play an important role in bread‐making technology since they determine how the dough behaves during mechanical handling and baking. In our study, the higher percentage of water absorption in doughs enriched with WGH might be attributed to the higher water‐binding ability of the hydrolysates compared to wheat flour. Additionally, considering that despite increased values for DDT, WGH at 4% also resulted in higher dough stability, we concluded that the hydrolysates have strengthened the gluten network rather than physically hindering it. Numerous studies have shown that when proteins are hydrolyzed by enzymes, the number of polar groups and hydrophilicity increases, accompanied by reduction in the molecular mass chains, and changes in molecular structure. Previous researchers have observed a weakened gluten network in dough when exogenous hydrolysates were added, which is in contrast with our results. We hypothesized that this contradiction might be due to the origin of the added exogenous hydrolysates (maize germ, lima bean, and cowpea vs. gluten). The positive effect of the WGH added in our study could be linked to the modification of the cross‐links and interactions between wheat proteins and the added WGH, in accordance with previous studies that suggest enzymatic hydrolysis of gluten can improve its solubility, foaming, and emulsifying capacities (Franco‐Miranda et al., 2017; Gabler & Scherf, 2020; Karimi et al., 2021; Wang et al., 2007; Zouari Ellouzi et al., 2014).

### 3.4 Effect of gluten hydrolysate levels on specific volume, moisture content, and color of the breads.

Bread specific volume (SV) was affected by the different amounts of WGH, demonstrating an increase in SV after the addition of hydrolysates (Table 2). The highest and second highest SV values were observed in the breads enriched with 4% and 2% hydrolysates, respectively. The control bread had the smallest volume, and the SV of the 1% bread sample was only slightly higher (*p* < .05). These results were in contrast with the findings of Fitzgerald et al. (2014) who reported that 4% hydrolysate obtained from *Palmaria palmata* protein had a reducing effect on bread SV and Karimi et al. (2021) who observed no significant effect on bread SV after the incorporation of 4% maize germ protein hydrolysate. As a result of these comparisons, we concluded that the increase in bread SV with higher inclusion levels of WGH was probably due to the source of the obtained hydrolysates and/or lack of competition between the added hydrolysates and wheat starch for moisture. Also, considering the numerous observations in literature about gluten hydrolysates' emulsifying properties, it could be suggested that gluten hydrolysate might increase bread volume by binding to protein hydrophobic surfaces, resulting in a strong protein network (Gabler & Scherf, 2020; Zouari Ellouzi et al., 2014). Regarding moisture content, measurements were carried out on days 1 and 4 of the storage period (Table 2). In comparison to the other bread samples, the bread with 4% hydrolysate had a significantly higher moisture content on day 1 of the storage, and the addition of hydrolysates did not significantly affect the other treatments. After 4 days, it was found that the moisture content of all bread samples had decreased, possibly due to staling that occurs with time, but the decreases in the moisture contents of the 4% and 2% bread samples were significantly lower than those of the control and the bread sample with 1% WGH.

**TABLE 2:** Specific volume, moisture content, and color analysis of wheat breads containing WGH at different levels.

	Wheat dough (control)	1% WGH	2% WGH	4% WGH
Specific Volume (mL/g)	2.90 ± 0.03^d^	3.10 ± 0.02^c^	3.25 ± 0.03^b^	3.40 ± 0.03^a^
Moisture content (%) at day 1	37.26 ± 0.62^b^	38.02 ± 0.15^b^	38.44 ± 0.23^b^	39.60 ± 0.26^a^
Moisture content (%) at day 4	28.29 ± 0.28^c^	28.95 ± 0.31^c^	33.71 ± 0.77^b^	36.28 ± 0.83^a^
Color (Crust)				
L*	59.45 ± 1.28^a^	51.27 ± 1.24^b^	49.65 ± 0.45^b^	43.93 ± 0.47^c^
a*	15.75 ± 0.56^c^	19.67 ± 0.55^b^	20.38 ± 0.48^b^	24.46 ± 0.76^a^
b*	31.67 ± 0.65^b^	32.47 ± 0.77^b^	32.68 ± 0.61^b^	34.56 ± 0.85^a^
Color (Crumb)				
L*	79.12 ± 0.25^a^	76.22 ± 0.45^b^	75.12 ± 0.57^b^	72.85 ± 0.23^c^
a*	1.47 ± 0.10^b^	1.64 ± 0.16^b^	1.68 ± 0.12^b^	2.45 ± 0.23^a^
b*	22.48 ± 0.38^c^	23.74 ± 0.44^b^	23.93 ± 0.32^b^	25.68 ± 0.23^a^

*Note*: The results are mean values of experiments carried out in triplicate. Different letters in a row indicate significant differences among samples (*p* < .05).

Color parameters of the crust and crumb of the breads were determined on day 1 post‐baking, and L*, a*, and b* values were measured (Table 2). Increasing concentrations of WGH influenced the luminosity (L*) of the crust, with the 4% bread obtaining the lowest L* value and the control bread the highest. Regarding a* value of the crust, lowest value was obtained for the control bread and the highest for the bread with 4% WGH, indicating a tendency for red coloration by the addition of WGH. Bread samples enriched with 2% and 1% WGH did not differ statistically in L* and a* values of the crust. In relation to b* value of the crust, only the 4% bread was statistically different from the other treatments, implying a more yellowish tone for this particular bread sample. In terms of crumb, in a trend similar to crust, L* decreased with increasing levels of added hydrolysates. For a* value of the crumb, only the bread with 4% WGH displayed values significantly higher than those of the other samples. As for b* value of the crumb, the 4% bread had the highest value and the control the lowest, implying more yellowish‐colored crumbs with increasing levels of hydrolysates, and no statistically significant difference was detected between breads enriched with 2% and 1% WGH. Consumers' acceptance level and purchase desire are largely determined by color attributes of products. According to our findings, WGH resulted in the development of more brown color and reddish/yellowish tones in the bread samples, possibly due to the involvement of the gluten hydrolysates and peptides with free amino groups in the Maillard reaction and caramelization browning (Karimi et al., 2021; Lafarga et al., 2016; Zhao et al., 2021).

### 3.5 Texture profile analysis of the breads enriched with ficin‐hydrolyzed gluten

The TPA values of the breads with added WGH on days 1 and 4 post‐baking are shown in Table 3. Among various textural characteristics, hardness is commonly considered the most important quality attribute of bread products (Zhao et al., 2021). On day 1, we observed a significant (*p* > .05) decrease in bread hardness at higher levels of WGH incorporation (2% and 4%), whereas no statistical difference was found between the bread sample with 1% WGH and the control bread. On day 4, hardness values increased for all bread samples; however, a much lower increase was observed in breads enriched with 2% and 4% hydrolysates in comparison with other treatments. In terms of cohesiveness, the bread formulation with 4% WGH presented the most cohesive crumb, followed by the bread sample with 2% WGH. Statistically, the control and the bread sample with 1% WGH had similar cohesiveness values. On day 4 of storage time, cohesiveness values decreased for all bread samples. However, the bread samples with 4% and 2% WGH maintained higher cohesiveness in comparison with the control and the bread sample with 1% WGH. Regarding springiness, in accordance with the aforementioned TPA values, the control and the 1% bread demonstrated higher springiness qualities compared to the bread samples containing 4% and 2% WGH. Affected by the storage time, springiness values for bread samples with 4% and 2% WGH were still lower than those of the control and the bread with 1% WGH on day 4.

**TABLE 3:** Textural profiles analysis of breads enriched with different gluten hydrolysate levels.

Treatment	Storage time (days)	Hardness (N)	Cohesiveness	Springiness (mm)	Chewiness (mJ)
Control	1	7.30 ± 0.21^d^	0.67 ± 0.00^c^	6.91 ± 0.01^b^	30.38 ± 0.51^d^
	4	17.90 ± 0.30^a^	0.63 ± 0.01^d^	7.03 ± 0.02^a^	72.09 ± 2.12^a^
1% WGH	1	7.11 ± 0.15^d^	0.67 ± 0.01^c^	6.81 ± 0.02^c^	29.96 ± 0.15^d^
	4	13.21 ± 0.70^b^	0.64 ± 0.01^d^	6.98 ± 0.03^a^	54.51 ± 2.70^b^
2% WGH	1	4.38 ± 0.69^e^	0.69 ± 0.01^b^	6.73 ± 0.02^d^	23.56 ± 1.05^e^
	4	11.34 ± 0.51^c^	0.66 ± 0.01^c^	6.92 ± 0.02^b^	44.24 ± 2.79^c^
4% WGH	1	3.14 ± 0.90^e^	0.72 ± 0.01^a^	6.45 ± 0.01^f^	17.38 ± 0.80^f^
	4	10.56 ± 0.35^c^	0.68 ± 0.01^b^	6.64 ± 0.02^e^	41.32 ± 2.51^c^

*Note*: Results are shown as mean ± standard deviation. Different letters in a column indicate statistically significant differences among samples (*p* < .05).

These results were in accordance with the values obtained for specific volume and moisture content, as previously discussed. Researchers have suggested that changing the type and nature of the proteins in wheat bread and increasing the hydrolysate content will result in a greater change in the gluten network (Madruga et al., 2020). Overall, our study showed that higher amounts of WGH produced by ficin protease resulted in decreasing values for hardness, springiness, and chewiness properties, as well as an increasing tendency with respect to cohesiveness. These findings could suggest that ficin‐hydrolyzed gluten, at certain amounts, possesses anti‐staling properties, presumably by creating an extensible protein network and maintaining softer bread structures (Zhao et al., 2021). It is also likely that the addition of hydrolysates might have prevented starch retrogradation, which is associated with staling during storage time and other compositional changes that affect texture and consumer acceptance. The formation of ionic and hydrogen bonds caused by the hydrability of negatively charged peptides may inhibit the long‐term retrogradation of amylopectin crystallites, one of several anti‐retrogradation mechanisms suggested for hydrolysates (Franco‐Miranda et al., 2017; Karimi et al., 2021).

### 3.6 Evaluation of in vitro antioxidant activity

#### 3.6.1 Antioxidant capacity of the non‐digested breads

Taking into account that a complex mixture of compounds with varying polarities contributes to the antioxidant properties of food matrices (Gawlik‐Dziki et al., 2014), DPPH, ABTS radical scavenging, and Fe^2+^ chelating assays were used to evaluate the antioxidant capacity of the bread samples at different levels of ficin‐generated gluten hydrolysates (Table 4). According to our findings, all samples containing hydrolysates displayed higher antioxidant activity than the control, with increasing concentrations of hydrolysates enhancing the antioxidant capacity of the extracts obtained from the breads (*p <* .05). The highest antioxidant values corresponded to the bread with 4% added gluten hydrolysate, as determined by all three methods; and the bread with 1% hydrolysate presented values more similar to the control in comparison with the other treatments. Additionally, the ABTS RSA method yielded results with greater values in comparison with the DPPH RSA and Fe^2+^ chelating techniques. These findings were in accordance with publications that suggest hydrolysates improve antioxidant activity, as similar results were observed by Karimi et al. (2021) who worked with maize germ protein hydrolyzed with Alcalase and Franco‐Miranda et al. (2017) who experimented with Lima bean and cowpea hydrolysates obtained by Pepsin and Pancreatin, incorporating the resultant hydrolysates into wheat breads and *concha*‐type Mexican sweet breads, respectively.

**TABLE 4:** Effect of WGH levels on bread in vitro antioxidant activity before and after digestion.

Treatments	Wheat dough (control)	1% WGH	2% WGH	4% WGH
*Non‐digested*				
DPPH	622.97 ± 11.15^d^	765.01 ± 13.53^c^	958.93 ± 12.74^b^	1225.22 ± 26.63^a^
ABTS	727.98 ± 27.25^d^	854.10 ± 29.45^c^	1091.96 ± 38.30^b^	1445.64 ± 37.58^a^
Fe^2+^	376.28 ± 14.03^d^	424.71 ± 13.66^c^	493.11 ± 18.66^b^	688.76 ± 21.06^a^
*Digested*				
DPPH	685.50 ± 16.06^d^	928.32 ± 15.29^c^	1236.74 ± 17.83^b^	1764.48 ± 13.96^a^
ABTS	10130.30 ± 15.01^d^	10,711.33 ± 19.00^c^	12,079.67 ± 20.74^b^	15,843.08 ± 18.38^a^
Fe^2+^	8871.22 ± 19.51^d^	9062.53 ± 21.68^c^	9867.72 ± 24.66^b^	11,346.43 ± 15.61^a^

*Note*: DPPH, ABTS radical scavenging (μmol Trolox eq/mg sample), and Fe^2+^ chelating (μmol EDTA eq/mg sample) activities of non‐digested and digested bread samples containing different levels of gluten hydrolysates produced by ficin; The results are mean values of experiments carried out in triplicate. Different letters in a row indicate significant differences among samples (*p* < .05).

It is well known that by hydrolyzing proteins enzymatically, antioxidant amino acids are exposed to greater levels, resulting in higher antioxidant activity. Furthermore, a variety of parameters affect the antioxidant activity of hydrolysates and peptides, such as the raw material utilized as the protein source, the type of protease and its specificity, the resultant degree of hydrolysis, and the nature of the generated peptides (including molecular weight, amino acid composition, and sequence). Our study indicated that the gluten hydrolysate obtained by ficin had antioxidant properties, suggesting that the obtained hydrolysate sample contained peptides and amino acids that could act as electron or hydrogen donors, thereby terminating a radical chain reaction by converting free radicals to stable products (Elmalimadi, Jovanovića, et al., 2017; Elmalimadi, Stefanovića, et al., 2017; Seyedain‐Ardabili et al., 2023; Wang et al., 2007).

According to reports, acquiring a strong radical scavenging activity generally requires a suitable degree of hydrolysis. Our SDS‐PAGE results showed that the ficin‐hydrolyzed gluten contained higher levels of peptides with medium to low molecular masses (Mr ≤ 15 kDa) in comparison with the control gluten sample, and these results were consistent with other studies that claimed radical scavenging activity and Fe^2+^ chelating ability significantly increased with the progression of wheat gluten hydrolysis and achieving a suitable high DH due to higher levels of low MW peptides that could interact with radicals more efficiently. These studies have also suggested that low‐molecular‐weight peptides tend to possess a higher negative charge (carboxyl group)‐to‐mass ratio than their larger counterparts, making them more efficient at forming complexes with metal ions. Furthermore, a difference in radical solubility and diffusivity in the reaction medium could explain the differences observed between ABTS+ and DPPH radical scavenging activities. In contrast with DPPH radicals, which can only dissolve in organic media, ABTS+ radicals can dissolve in both organic and aqueous solutions (Cotabarren et al., 2019; Elmalimadi, Jovanovića, et al., 2017; Karimi et al., 2021; Nikoo et al., 2019; Shahi et al., 2020).

There aren't many studies on the usage of protein hydrolysates in bakery products owing to a number of challenges, such as hydrolysate inclusion levels, stability, and alteration of bioactivity through processing and baking conditions (Franco‐Miranda et al., 2017; Karimi et al., 2021; Madruga et al., 2020). Based on our results, the added hydrolysates at all levels maintained a degree of stability despite heating conditions, enhancing antioxidant capacity, which could be due to the presence of greater amounts of low‐molecular‐weight (LMW) peptides. These LMW peptides would be more easily accessed by the oxidant system and may also interact with the bread matrix components to generate new bioactive compounds. Also, researchers have found that the browning reaction might enhance the antioxidant qualities. According to several reports, Maillard reaction products, particularly those that form in the later stages of the reaction, have the ability to scavenge free radicals and could contribute to antioxidant properties (Cian et al., 2011; Karimi et al., 2021; Meral & Erim Köse, 2019).

#### 3.6.2 Effect of in vitro digestion on bread antioxidant capacity

Given that bioactive compounds are sensitive to a variety of environmental parameters, in vitro gastrointestinal digestion is frequently utilized to investigate changes in the bioactivity of the foods enriched with such compounds and their potential impacts on human health (Zhang et al., 2022). In our study, the antioxidant activity of the enriched bread samples was evaluated using an in vitro simulation model of human digestion system (Table 4). Our findings revealed that after the digestion process, bread antioxidant values obtained through all three methods of antioxidant assessment increased, with the bread sample containing 4% WGH exhibiting the highest antioxidant activity and the control bread the lowest, which could lead to the conclusion that digestion in vitro has released more antioxidant compounds from both hydrolysates and the base product (wheat bread). A direct correlation was found between the antioxidant values and the percentage of the added ficin‐hydrolyzed gluten, supporting the theory that the bioactive components added not only were stable against pH changes and enzymatic degradation but may also have been further functionalized by digestion to chelate Fe^2+^ and scavenge ABTS and DPPH radicals.

A variety of factors might have caused this rise in antioxidant capacity. The action of digestive enzymes and bile salts on the food matrix may be responsible for the elevated antioxidant levels, leading to the release of more bioactive chemicals, peptides, and amino acids. The digestive enzyme pepsin has a high affinity for aromatic residues, resulting in a relatively low level of digestion, while pancreatin possesses a broader specificity, as it consists of endopeptidases and exopeptidases. Larger peptides present in the added gluten hydrolysates might have been further hydrolyzed by these gastrointestinal enzymes, generating smaller peptides with higher bioactivity. According to literature, low‐molecular‐weight peptides (<3 kDa) with higher hydrophobicity generally exhibit stronger radical scavenging abilities, reacting more effectively with both ABTS+ and DPPH free radicals. However, because there are not many negatively charged amino acid residues in these shorter peptides, they might not display good metal chelating activity.

Some studies have proposed that chelating metals with high‐molecular‐weight fractions (5–10 kDa) might be more effective due to the synergistic effects of higher number of amino acid residues, and increased quantities of carboxylic and amino group in branches of the basic and acidic amino acids may help enhance metal ion binding and removal (Karimi et al., 2021; Kashyap et al., 2022; Seyedain‐Ardabili et al., 2023; Zhang et al., 2020, 2022). Researchers have also hypothesized that upon GI digestion, hydrolysates might eventually produce peptides of similar sizes. In this instance, variations in the peptide sequences could be primarily responsible for the differences in the antioxidant activity. Furthermore, free amino acids such as tryptophan, histidine, phenylalanine, and tyrosine could play major roles in antioxidant activity through proton/electron transfer mechanisms. The chemical and structural alterations of antioxidant compounds during in vitro digestion, presumably as a result of synergistic interactions among bioactive components and/or food constituents, could be another possible explanation for the increased antioxidant values after digestion (Corrochano et al., 2019; Pavez‐Guajardo et al., 2020; Zhang et al., 2020).

#### 3.6.3 Sensory evaluation

The results of sensory analysis conducted on the enriched bread samples are presented in Table 5. In terms of external parameters, the scores acquired for the crust colors of the breads containing 2% and 4% hydrolysates were significantly different from those of the breads enriched with 0% and 1% hydrolysates. As for the internal characteristics, the aroma score of the 4% bread sample was significantly higher than all other treatments. Also, the bread samples containing 2% and 4% WGH both received significantly higher scores for chewability and texture parameters, in comparison with the other treatments. Regarding taste, the 4% bread scored the lowest, but the obtained value did not statistically differ from those of the other bread samples. Some studies have observed a bitter flavor and loss of aroma in association with the added hydrolysate and peptide contents from protein sources other than gluten, which they hypothesized could be related to the generation of new Maillard products with different aroma compounds owing to the inclusion of protein hydrolysates containing various peptides and amino acid residues in bread formulations (Fitzgerald et al., 2014; Karimi et al., 2021). Therefore, it might be noteworthy that our findings showed a distinctive pattern among breads in relation to sensory characteristics, with the enriched bread samples scoring higher than the control bread. Hence, it can be concluded that the added levels of ficin‐produced gluten hydrolysates influenced the overall sensory preference in a favorable way.

**TABLE 5:** Sensory evaluation results of breads enriched with different levels of ficin‐hydrolyzed gluten.

Bread	Perfect score	Control	1% WGH	2% WGH	4% WGH
*External parameters*					
Volume	10	9.10 ± 0.40^a^	9.13 ± 0.30^a^	9.30 ± 0.19^a^	9.50 ± 0.23^a^
Color of crust	8	6.83 ± 0.16^b^	6.85 ± 0.14^b^	7.24 ± 0.10^a^	7.48 ± 0.18^a^
Symmetry of form	3	2.68 ± 0.12^a^	2.71 ± 0.15^a^	2.74 ± 0.13^a^	2.80 ± 0.14^a^
Evenness of bake	3	2.50 ± 0.25^a^	2.72 ± 0.25^a^	2.76 ± 0.22^a^	2.80 ± 0.18^a^
Character of crust	3	2.65 ± 0.23^a^	2.66 ± 0.23^a^	2.69 ± 0.26^a^	2.74 ± 0.20^a^
Break and sherd	3	2.70 ± 0.30^a^	2.67 ± 0.35^a^	2.74 ± 0.16^a^	2.80 ± 0.20^a^
*Internal parameters*					
Grain	10	9.35 ± 0.35^a^	9.23 ± 0.24^a^	8.89 ± 0.34^a^	8.73 ± 0.25^a^
Color of crumb	10	9.24 ± 0.15^a^	9.21 ± 0.10^a^	9.25 ± 0.16^a^	9.20 ± 0.19^a^
Aroma	10	9.15 ± 0.16^b^	9.09 ± 0.20^b^	9.25 ± 0.13^b^	9.57 ± 0.12^a^
Taste	15	14.17 ± 0.30^a^	14.00 ± 0.33^a^	14.13 ± 0.29^a^	13.75 ± 0.53^a^
Chewability	10	8.57 ± 0.17^b^	8.62 ± 0.15^b^	9.07 ± 0.16^a^	9.23 ± 0.20^a^
Texture	15	13.61 ± 0.29^b^	13.73 ± 0.18^b^	14.25 ± 0.22^a^	14.43 ± 0.21^a^
*Staleness of bread*					
At day 1 of storage		1.00 ± 0.00^a^	1.00 ± 0.00^a^	1.00 ± 0.00^a^	1.00 ± 0.00^a^
At day 4 of storage		4.60 ± 0.16^a^	4.21 ± 0.21^a^	3.50 ± 0.29^b^	3.15 ± 0.25^b^

*Note*: Different letters in a row indicate significant differences among samples (*p* < 0.05).

Regarding staleness of the breads, no significant differences were observed among different treatments on day 1 of storage time. On day 4, the breads containing 2% and 4% ficin‐generated WGH received scores that were notably different from those of the other treatments (*p* < .05) and were perceived as less stale, as presented in Table 5 by lower scale values (3 = slightly fresh, 4 = slightly stale and 5 = stale), indicating that in accordance with the results obtained from the textural analysis of the bread samples, the gluten hydrolysates added up to 4% resulted in anti‐staling properties in bread during storage.

## 4 CONCLUSION

In conclusion, the addition of ficin‐hydrolyzed wheat gluten up to 4 g per 100 g flour positively influenced the technological and physical–chemical characteristics of the wheat bun breads, demonstrated anti‐staling properties during storage, and received satisfactory responses from the sensory evaluation panel. The obtained in vitro results showed that the enriched bread products possessed DPPH, ABTS radical scavenging, and Fe^2+^ chelation abilities that increased in response to higher levels of hydrolysate inclusion, and were resistant to the bread preparation and baking process. Furthermore, these antioxidant activities were enhanced by the simulated gastrointestinal digestion conditions. Based on our overall findings, wheat gluten hydrolysates produced by ficin could be used to produce wheat breads with enhanced physicochemical, nutritional, and biological quality, beneficial to health.

## AUTHOR CONTRIBUTIONS

**Mojan Seyedain‐Ardabili:** Conceptualization (lead); data curation (lead); formal analysis (lead); funding acquisition (equal); investigation (lead); methodology (lead); project administration (equal); resources (lead); software (lead); supervision (supporting); validation (lead); visualization (lead); writing – original draft (lead); writing – review and editing (lead). **Mohammad‐Hossein Azizi:** Conceptualization (supporting); data curation (supporting); formal analysis (supporting); investigation (supporting); methodology (supporting); project administration (supporting); resources (supporting); supervision (lead); validation (supporting); writing – review and editing (supporting).

## CONFLICT OF INTEREST STATEMENT

The authors confirm that they have no conflicts of interest with respect to the work described in this manuscript.

## Data Availability

All data generated or analyzed during this study are included in this published article.
